# Acute Dosing Strategy with Vistula Tart Cherries for Recovery of Strenuous Exercise—A Feasibility Study

**DOI:** 10.3390/nu16162709

**Published:** 2024-08-15

**Authors:** Emma Squires, Ian H. Walshe, Alex Dodd, Edward Broadbelt, Oliver Hayman, Malachy P. McHugh, Glyn Howatson

**Affiliations:** 1 Faculty of Health and Life Sciences, Northumbria University, Newcastle upon Tyne NE1 8ST, UK; e.squires@northumbria.ac.uk (E.S.); ian2.walshe@northumbria.ac.uk (I.H.W.); mchugh@nismat.org (M.P.M.); 2 School of Cardiovascular and Metabolic Health, BHF Glasgow Cardiovascular Research Centre, College of Medical, Veterinary, and Life Sciences, University of Glasgow, Glasgow G11 6EW, UK; 3 Nicholas Institute of Sports Medicine and Athletic Trauma, New York, NY 10065, USA; 4 Water Research Group, North West University, Potchefstroom 2531, South Africa

**Keywords:** tart cherry, nutritional supplementation, muscle damage

## Abstract

Tart cherry (TC) consumption has become a popular nutritional strategy for recovery, particularly for the attenuation of markers associated with muscle damage. However, there are relatively few studies that have examined an acute dosing strategy. The aim of this pilot study was to explore the feasibility of using powdered Vistula TC for recovery following a bout of muscle-damaging exercise. Twenty-two recreationally active participants (mean ± SD age, stature, and mass were 23 ± 3 years old, 173 ± 10 cm, and 74 ± 17 kg, respectively) performed 40 (5 sets of 8 repetitions) maximal lengthening contractions of the elbow flexors. The participants were randomised to receive either a spray-dried TC extract or a calorie-matched placebo (12 TC, 10 placebo) for 4 days in total, starting on the day of exercise. Dependent measures of maximal voluntary contraction (MVC), muscle soreness (assessed via visual analogue scales; VAS), pain pressure threshold (PPT), range of motion (ROM), and upper arm limb girth were taken at baseline (pre), 24, 48, and 72 h post damaging exercise. There were significant changes over time among all the variables (MVC, VAS, PPT, ROM, and girth, *p* ≤ 0.014). There were no significant differences between the conditions for any of the variables (MVC, VAS, PPT, ROM, and girth, *p* > 0.3). The TC group did not recover at an accelerated rate compared to the placebo. This study provides initial insights into the use of powdered Vistula TC and its effect following strenuous (damaging) exercise bouts. Vistula TC did not improve recovery when taken acutely following a bout of damaging exercise to the elbow flexors.

## 1. Introduction

Exercise-induced muscle damage (EIMD) results in decreased force-generating capacity and range of motion, localized swelling, delayed onset of muscle soreness (DOMS), and the attenuation of increased intramuscular proteins and markers of inflammation in the blood, such as creatine kinase and C-reactive protein [1]. The damage occurs through a complex combination of mechanisms including structural damage to the muscle fibres as well as a secondary inflammatory response, which leads to the infiltration of neutrophils and macrophages to the affected area [2,3]. There is growing interest in the use of foods containing high levels of (poly)phenols in an attempt to reduce the EIMD response [4,5,6,7,8,9]. In particular, tart cherries (TCs), which are considered good sources of polyphenols and anthocyanins [10,11], have been extensively researched due to their benefits in exercise recovery and health. The benefits have been attributed to the properties of specific (poly)phenols, which are documented to reduce inflammation and reactive oxygen and nitrogen free radical production via the inhibition of the cyclooxygenase (COX-1 and COX-2) pathways [12,13]. 

Amongst the exercise recovery literature, Montmorency TCs have been documented to mitigate the loss of force production following EIMD [14,15,16,17,18,19,20], reduce the elevation of one or more markers of inflammation [14,18,19,21,22] and oxidative stress [17], and reduce DOMS [15,22,23,24]. Although studies on TCs have generally reported favourable effects on one or more indicators of EIMD, some studies have reported no beneficial effects [8,25,26,27,28], but no study (to the best of our knowledge) has shown a negative effect.

The majority of studies utilising Montmorency TCs in exercise recovery paradigms implement a loading phase: a period of ingestion prior to the damaging bout of exercise. Connolly, McHugh, and Padilla-Zakour [15] were the first to show that a proprietary TC juice blend was effective at reducing indices of pain and improved force recovery by providing two servings (12 oz bottle containing at least 40 mg anthocyanins) per day for 8 days, specifically 3 days prior, the day of, and 4 days post-exercise. Subsequently, studies have implemented consumption periods ranging between 3–20 days, with most including a loading phase prior to exercise ranging between 1 and 16 days. It is important to note that in team sports where starting players are sometimes confirmed as late as on the day of the fixture, it is not always feasible to provide a loading phase. To date, there is one study which has not provided a loading phase and instead started the supplementation on the day of exercise [28]. The authors reported no benefit from the consumption of TCs on markers of functional recovery or on subjective well-being. However, there are some studies that used a loading phase prior to exercise and observed no differences in EIMD markers between the TC and placebo groups [8,26]. Therefore, the results cannot be purely attributed to the dosing strategy.

A loading phase relies on the premise that there is a bioaccumulation of compounds. Although conducted in a rodent model [29], three weeks of supplementation with powdered Montmorency TCs showed that anthocyanins were found to be stored in the bladder, liver, kidneys, and brain. It is important to note that the study did not assess muscle storage. Moreover, following 10 days of Montmorency TC supplementation in humans, Wangdi, O’Leary, Kelly, Jackman, Tang, Dutton, and Bowtell [16] demonstrated increased protein expression of glutathione peroxidase 3, a marker of antioxidant activity, from within the *vastus lateralis* muscle. However, there has been no study investigating the bioaccumulation of anthocyanins in humans, so it is unclear how to optimise the supplementation period of TCs. Research investigating a dose–response relationship [30], using two doses of Montmorency TCs (30 and 60 mL concentrate), found (poly)phenol peaks in plasma concentrations at 1.5–2 h post-ingestion, with the lower dose returning to baseline by 8 h. In another dose–response study [31], there were observed differences between the doses, whereby plasma cyanidin-3-O-glucosiderutinoside concentrations were significantly different between the low and high doses 1 h post-ingestion, and the high dose was consistently greater throughout the rest of the time points. More recently, similar results were observed in a European variety of TC, Vistula TCs [32]. A low and high dose of Vistula TC powder induced different responses in the metabolome, with differences still being evident 8 h post-ingestion. Moreover, there was a transient increase in anthocyanin concentrations in plasma, for both the high and low doses, where peaks were observed 1 and 3 h post ingestion. Although investigated in different forms, Vistula TC extract presented higher concentrations of vanillic acid compared to the Montmorency variety [30,32]. Hence, it seems plausible that the acute consumption of Vistula TCs could alleviate EIMD symptoms as the polyphenols will be at their highest plasma concentration when some markers of inflammation and EIMD appear. To date, no study has utilised the European Vistula TC variety in an exercise recovery paradigm without a loading phase. Therefore, the aim of this study was to examine the efficacy of powdered Vistula TCs immediately after the completion of a damaging bout of exercise and for the subsequent recovery period on the markers of EIMD.

## 2. Materials and Methods

### 2.1. Participants

Twenty-two recreationally active participants (10 females) completed this study; their characteristics are displayed in Table 1. The participants were healthy and non-smoking with no history of cardiovascular, gastrointestinal, thyroid, or renal disease. The participants were also free from musculoskeletal injuries. Further exclusion criteria stipulated the participants were not to be highly trained or partake in structured resistance training. The health and training status were determined using screening questionnaires. Highly trained was defined as completing structured and periodized training and developing towards (within 20%) maximal or nearly maximal norms within their given sport [33]. The participants were asked to refrain from all exercise five days prior to and for the duration of testing. Furthermore, the participants were asked to refrain from non-steroidal anti-inflammatory drugs and nutritional supplements, such as ibuprofen, paracetamol, protein, and vitamins C and E.

### 2.2. Experimental Design

In a double-blind, randomised, placebo-controlled, parallel group design, participants were randomly assigned to consume either TC or placebo capsules, which were matched for calorific content. The participants reported to the laboratory on five occasions. This included a familiarisation visit (visit 1), followed by four visits on consecutive days (visits 2–5). Visit 2 consisted of one damaging bout of exercise and a baseline assessment of functional performance and perceptual variables. Visits 3–5 assessed the same functional and perceptual measures to assess the time course of recovery. All the test visits took place at the same time of day.

### 2.3. Damage Protocol

An isokinetic dynamometer (Biodex System 4, New York, NY, USA) was set up as recommended by the manufacturer to test the elbow flexors. The participant’s position was stabilised using a pelvic strap and two shoulder straps to minimise mechanical assistance from other body parts. The final position of the dynamometer seat and power head were recorded and replicated for each participant’s dominant arm to ensure consistency on subsequent days. The joint position and range of motion were monitored throughout by extracting data from the dynamometer to an analogue-to-digital converter (Micro 1401, CED, Cambridge, UK) to ensure that on return visits each participant was placed in an identical position (recorded on Spike2, v7.12; Cambridge Electronic Design, Cambridge, UK). In accordance with previous recommendations [34], the dynamometer readings were corrected for the gravity acting upon the limb mass. The dynamometer was set to an angular velocity of 30°/s^−1^ in the passive mode and the participants were instructed to resist maximally from full elbow flexion through the whole anatomical ROM until full extension had been achieved. Each participant completed 40 (five sets of eight) maximal lengthening contractions of the elbow flexors, with each set separated by 90 s rest. Previous research utilising a protocol of the same intensity but varying in volume—both lower and higher—successfully induced muscle damage [35]. Visual feedback from the dynamometer and standardised strong verbal encouragement from the investigator were given during the damaging exercise to encourage maximal effort throughout the protocol [35].

### 2.4. Supplementation

The participants consumed either a placebo or a TC spray-dried extract made from a variety of TC known as ‘Nadwiślanka’, also called Vistula cherries, in the form of capsules, twice per day (in the morning and evening) for a period of four days. The supplementation began on the day of exercise and continued for three days following the exercise bout. The dose (5.1 g of spray-dried extract contained 132.3 mg of anthocyanins expressed as cyanadin-3-glucoside measured via high-performance liquid chromatography and 611.9 mg of polyphenols measured via the Folin-Ciocalteu method, according to the manufacturers analysis) was determined by replicating similar volumes of the reported anthocyanin content from previous studies that used powdered TCs [22,23,36]. Previous studies have provided an average of 111 mg of total anthocyanins [22,23,36] and 991 mg of total polyphenols [22,23], for 10 days once per day [22,23] and 20 days twice per day [36].

### 2.5. Functional Performance and Perceptual Variables

At baseline, 24, 48, and 72 h after the damage protocol (visits 2–5), measures of functional performance and perceptual variables were used to assess the recovery after an isolated muscle damage protocol.

#### 2.5.1. Maximal Voluntary Contraction (MVC)

The participants performed a warm-up on the aforementioned dynamometer, which consisted of three submaximal contractions at 30, 50 and 70% of what the self-perceived MVC was. The MVC was determined at a joint angle of 45° of flexion from full extension, following an identical method that reported good repeatability (CV < 5%; [37]. Three isometric contractions were performed, each lasting ~3 s with 30 s rest between the repetitions. The peak torque was determined as the maximal torque generated over the three repetitions. The participants were given visual feedback via the dynamometer monitor to maximise the peak torque [38].

#### 2.5.2. Muscle Soreness

The muscle soreness of the participants’ elbow flexors was assessed via the use of a 200 mm visual analogue scale (VAS) and the pain pressure threshold (PPT) using a handheld pain algometer (Pain Test FPIX 50 Force One, Wagner Instruments, Greenwich, CT, USA) during visits 2–5. The participants rated their elbow flexor soreness during active flexion and extension of the elbow on the VAS; the far-left end point represented ‘no pain’ (0 mm) and the far-right end point represented ‘unbearably painful’ (200 mm). For the PPT measurements, the participants were seated upright, with their arms rested at 90 degrees from full flexion. The application of increasing pressure of the algometer was applied via the probe through a 1 cm diameter head at a continuous rate of 10 Ns^−1^ to the belly of the muscle until the participants indicated the pain to be intolerable. At this point, the force value (N) was recorded. Two measurements were taken (2 cm apart) at each site by the same investigator at baseline, 24, 48, and 72 h after completion of the damage protocol. The location of the measurements was marked with a semi-permanent pen in order to ensure consistency on consecutive days. The mean of the two measurements was used for data analysis (N).

#### 2.5.3. Limb Girth

An anthropometric tape measure (SciChem, Aberdeen, UK) was used to determine the bicep circumference while the arm was relaxed down by the participant’s side. The mid-bicep circumference was determined by locating the mid-way between the acromion process and the lateral epicondyle of the humerus. The mid-point was marked using a semi-permanent pen to ensure consistency on subsequent days. The same investigator took the average of two measurements, unless they were >5%, in which case, a third was taken and the average was taken from three measurements. This occurred for less than 10% of the total girth measurements taken.

#### 2.5.4. Range of Motion (ROM)

The joint ROM was determined by asking the participants to stand upright with their arms flexed at the shoulder and elbow. From this position, the participants were asked to fully extend the limb until they were unable to move further without experiencing discomfort. The elbow joint angle was determined using a goniometer and universal landmarks (the styloid process of the radius, the lateral epicondyle of the humerus, and the acromion process) to ensure correct alignment [39]. The landmarks were marked using a semi-permanent pen so the goniometer could be placed in the same way on consecutive days. The same investigator took the average of two measurements, unless they were >5%, in which case, a third was taken.

### 2.6. Data Analysis

A power calculation was performed for the primary outcome variable: MVC. Using the findings of previous studies that examined group differences in isometric strength [14,18], it was estimated that a ≥10% group difference (SD: 7.5%, based on % change from baseline data) would be required to detect significant changes. With a power of 0.80 and a two-tailed α level of 0.05, the estimated number of participants required was *n* = 9 per group.

All the statistical analyses were conducted using the Statistical Package for the Social Sciences (SPSS for Windows, version 28, SPSS Inc., Chicago, IL, USA) and the data were expressed as the mean ± standard deviation (SD). A 2 × 4 mixed model analysis of variance (ANOVA) was used to test for differences between all the dependent variables. Data analysis for the PPT, ROM, and MVC were conducted using the percentage change from the baseline values to account for individual variability. If significant time and/or interaction effects were observed, Fisher least significant difference post hoc tests were performed to locate where the differences occurred. Confidence intervals (CIs) of 95% were reported for the main effects. The significance was set at *p* < 0.05.

## 3. Results

The participants reported no side effects for either treatment, which were ingested easily and considered suitably palatable. All the variables showed a time effect (MVC, VAS, PPT, and ROM, *p* < 0.001, girth, *p* = 0.014), indicating that the protocol was effective at inducing muscle damage. The work performed was similar between the groups during the damage protocol (TC 2090 ± 887 J vs. placebo 1902 ± 1174 J, *p* = 0.69).

**Figure 1:**
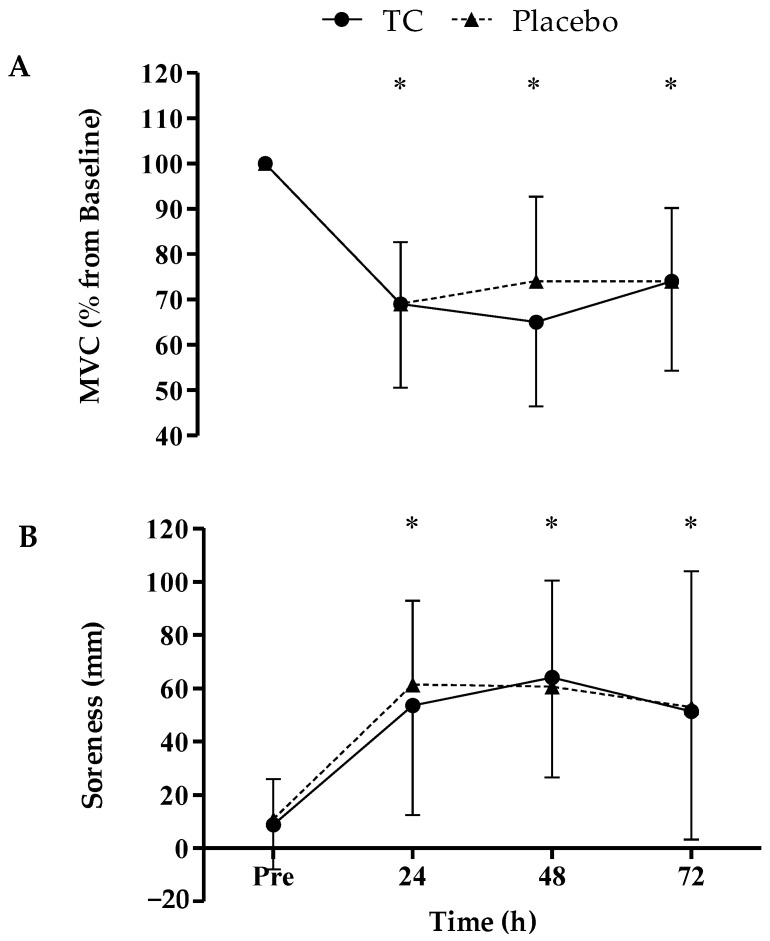
Changes in MVC (**A**) and soreness (**B**) in response to TC (*n* = 12) and placebo (*n* = 10) supplementation. Values are mean ± SD; * indicates a significant time effect from baseline (*p* < 0.001).

The MVC (Figure 1A) showed a time effect, whereby each time point was significantly decreased from the baseline (*p* < 0.001, CI pre 23.83–38.56, 24 h 22.13–38.77, 48 h 17.49–33.78, 72 h −38.56–−23.83); however, there were no time × treatment interactions (F_(1,2.0)_ = 1.047, ES = 0.5, *p* = 0.361) and no differences between the groups (F_(1,2.0)_ = 0.175, ES = 0.009, *p* = 0.68, CI −13.39–8.92). Similarly, soreness (Figure 1B) showed a time effect whereby each time point was significantly increased from the baseline (*p* < 0.001, CI pre −64.64–−34.29, 24 h −74.15–−38.15, 48 h −72.18–−25.07, 72 h 34.29–64.64); however, there were no time × treatment interactions (F_(1,1.7)_ = 0.527, ES = 0.026, *p* = 0.57) and no differences between the groups (F_(1,1.7)_ = 0.031, ES = 0.002, *p* = 0.862, CI −28.43–33.67).

The PPT, limb girth, and ROM, as displayed in Table 2, showed no time × treatment interactions (F_(1,2)_ = 1.767, ES = 0.081, *p* = 0.184, F_(1,1.9)_ = 0.239, ES = 0.012, *p* = 0.783 and F_(1,1.9)_ = 0.910, ES = 0.044, *p* = 0.405, respectively) and no differences between the groups (F_(1,2)_ = 1.339, ES = 0.063, *p* = 0.261, CI −24.12–6.91, F_(1,1.9)_ = 0.191, ES = 0.009, *p* = 0.667, CI −3.66–5.60 and F_(1,1.9)_ = 1.263, ES = 0.059, *p* = 0.274, CI −7.72–2.32, respectively).

## 4. Discussion

This is the first study to evaluate the effects of a powdered Vistula TC variety on recovery indices following a bout of damaging exercise without a loading phase. Firstly, the bout of maximal eccentric exercise successfully caused muscle damage in both groups; however, powdered Vistula TC capsules without a loading phase did not result in accelerated recovery. The intervention was palatable and well tolerated, and there were no adverse effects reported.

In the present study, there were no differences between the groups for the recovery of strength following eccentric exercise (Figure 1). Much of the previous research has demonstrated the efficacy of Montmorency TCs as an intervention to mitigate strength loss or enhance recovery rates following strenuous exercise [14,15,16,17,18,19,20,40]. Although, there are a few studies that showed no beneficial effects [8,25,26,27,28]. The most notable difference between the present study and the studies that found TCs to be effective is the variety of cherries used; previous studies exclusively used the Montmorency cultivar, whereas the present study used the Vistula cultivar. In addition, the majority of studies used a loading phase, whereas only one study did not implement a loading phase [28], which also showed no differences between groups. A number of methodological differences exist between studies. Besides the variety and mode of delivery (capsules), it is well-known that the variety and indeed batch of TCs influence the phytochemical properties [41,42] and potentially the absorption and anti-inflammatory/antioxidant effects of the product. Although, plasma concentrations of polyphenols have been observed to be similar in dose–response studies of both Montmorency and Vistula TCs [30,31,32]. Furthermore, the present study utilised a laboratory-based, eccentric contraction-focused protocol that was designed to maximise EIMD. Previous work used a competitive soccer match [28], where there could potentially be a plethora of confounding factors influencing the results, not least the use of other recovery strategies. Moreover, the extent of muscle damage was vastly different, with the present study inducing moderate to severe levels of EIMD (≥20% loss in muscle function and/or ≥2 d for full recovery; Paulsen, et al. [43]), whereas the professional footballers experienced relatively mild EIMD (<6%). Despite the methodological differences, it is crucial to acknowledge that there is currently no evidence to support why the consumption of TCs for several days prior to exercise yields greater benefits compared to an acute strategy, albeit it was not successful in this instance. Moreover, a rationale for such a dosing strategy has yet to be elucidated. A previous study [29], conducted on rodents, demonstrated the bioaccumulation of polyphenol compounds in tissues after prolonged Montmorency TC ingestion (three weeks). These data suggested that a loading phase could be more beneficial, assuming the findings can be translated to humans. Notwithstanding, this study did not compare whether similar outcomes could be achieved with a single dose of TCs or if a loading phase was necessary. Conversely, bioavailability studies [30,31,32] indicated that small volumes of active phenolic compounds, suspected to modulate post-exercise inflammation and oxidative stress, are detectable at least 8 h after ingestion of Montmorency TCs. Moreover, the pharmacokinetic data revealed that polyphenol concentrations in TCs peak in plasma 1–2 h after ingestion. Thus, it is feasible that a post-exercise dose could be more efficacious as polyphenols reach their highest concentrations in the body when inflammation and EIMD are elevated. Therefore, this highlights an essential question for future research to examine the destination of polyphenols following consumption. It is clear that they increase acutely and can be excreted [44], but it is unclear if tissues, particularly skeletal muscle, absorb and store these compounds during a loading phase or if they induce significant biochemical adaptations, such as the upregulation of the endogenous antioxidant pathways.

The lack of effect that powdered Vistula TCs had on soreness and PPT agrees with the majority of previous research, albeit in the Montmorency variety [14,16,17,18,19,28]. However, some studies have shown that TCs showed an improvement regarding perceived pain [15,23,36]. Furthermore, similar to the present study, both Connolly, McHugh, and Padilla-Zakour [15] and Kastello, Bretl, Clark, Delvaux, Hoeppner, McNea, and Strauss [36] induced muscle damage in the elbow flexors. A similar pattern occurred whereby pain peaked at 24 h in the TC group and subsequently declined compared to the placebo group, where pain continued to increase and peak at 48 h [15]. As such, it is feasible to assume the lack of group effect might be explained by the differences between these studies; as previously discussed, the ingestion period is notably different as well as the ingestion form, i.e., powdered vs. juice/concentrate. Relatively few studies have used powdered Montmorency TCs [22,23,40,45]. One study used a Montmorency TC paste in capsules [36]. Of note, only one study using powdered Montmorency TCs demonstrated a reduction in strength loss following EIMD [40]. However, the evaluation of functional recovery relied on the hand-grip strength, which did not assess the recovery of damage given that the protocol involved 60 repetitions of barbell back squats at 80% 1RM. Furthermore, there were no differences between the groups in vertical jump performance, and the values remained unchanged across all time points, which is unexpected considering the nature of the damage protocol. Although no differences were observed in strength loss, powdered Montmorency TCs have been effective at reducing DOMS [23] and inflammation [22]. Nevertheless, the majority of the efficacy has been observed when using a Montmorency TC juice or concentrate [14,15,16,17,18,19,20,21]. Therefore, it appears that the use of powdered TC products to attenuate strength losses might not be as pronounced as that of other analogues, despite reports of higher anthocyanin and polyphenol levels in powdered/frozen cherries compared to concentrates [11]. Future studies could, however, explore a loading phase with a powdered analogue. It is worth noting that caution is warranted when interpreting the anthocyanin and polyphenol content per dose as analytical chemistry methodologies can vary between studies. Moreover, some studies [19,21,28] have reported phytochemical content based on previously published data and potentially different supplement batches, making it challenging to ascertain the actual content.

Significant reductions in the ROM and increases in the limb girth were observed across all time points compared to the baseline, although no differences were noted between the treatments. Few studies in the existing literature have assessed the ROM [8,15] and limb girth [36], and our findings align with these previous studies, indicating that TC supplementation did not affect the girth or ROM following strenuous exercise, regardless of the form of TC product or whether a loading phase was implemented. In addition, it seems that both the ROM and girth are unaffected by TCs, independent of whether TCs attenuated strength loss or positively influenced any other marker or recovery.

It might be expected that TCs would be more effective when there is a greater level of force loss, as this would indicate greater muscle damage and likely greater levels of inflammation and oxidative stress, both of which TCs are purported to mitigate. Yet, in the present study, there was a loss of strength comparable to previous studies that showed TCs to benefit recovery [16]. Differences in the training status of the participants and the modality of exercise might play a role in the effectiveness of TCs. Given the heteroscedasticity of the data, it is conceivable that the training was varied despite efforts to recruit a homogenous group [46]. Although, previous studies in different exercise modalities have shown Montmorency TCs to be beneficial within a spectrum of the cohorts’ training status [14,16,17,18,19,21], though not necessarily in the same study. There seems to be greater benefit in studies that have induced high metabolic stress [9], which was not present in this study due to the nature of the exercise protocol. In a recent meta-analysis, TCs were found to reduce markers of inflammation (small effect for both CRP and IL-6); this effect was greater when the exercise bout was metabolically challenging, such as long-distance running. It should be noted that these studies were not free from mechanical stress either due to repeated eccentric loading of the lower limbs [14,21,47]. Exercise modalities with a high metabolic component might prompt a systemic inflammatory response, and an increase in oxidative stress might occur [18,21]. It has been previously proposed that anthocyanins in TCs could mediate inflammation due to inhibition of the prostaglandin enzymes [48]. Therefore, it is plausible that TCs could be more suited to facilitate recovery that incorporates a high metabolic demand. However, there have been studies that have shown beneficial effects on recovery indices and no differences between the groups for indirect markers of inflammation [16,17,20].

A potential limitation of the present study is the lack of a dietary control and/or knowledge of the participants’ habitual diets. Several previous studies [8,21,36] have restricted participants’ dietary intake by excluding foods that could be high in polyphenols. However, this might lead to an overestimation of the intervention effects. Furthermore, it is plausible that this aspect did not influence the outcome as previous research has shown no differences between treatment groups, even with restrictions on the participants’ dietary intake [8,36]. However, it is an important question for future researchers to explore what benefit an intervention might have if nutrients high in polyphenols are consumed habitually, and for that, you would need an in-depth record of the participants’ habitual diets. Future studies could combine food diary intake with measurements of total antioxidant status, which would offer an in-depth understanding of both their polyphenol intake and body concentrations. Moreover, it is acknowledged that this study did not take any measurements of plasma polyphenol or anthocyanin content; therefore, the effects of the intervention cannot be conclusively attributed to the presence or absence of these functional compounds. However, the current study builds on a previous study using the same intervention [32], which demonstrated a plasma increase in these phytochemicals following acute supplementation. One other consideration is the dose consumed relative to the body mass and subsequent bioavailability differences that might be present. For example, a lower body mass might have greater plasma availability of the compounds, whereas heavier participants might be lower. Despite these considerations, this study provides an ecologically valid dosing strategy that is the same [32] or similar [22,23] to previous interventions that have had observed effects. 

## 5. Conclusions

This feasibility study provides initial insights into the use of a powdered Vistula TC product and its effect when consumed acutely on the day of a strenuous (damaging) exercise bout and during an initial recovery period. It is the first study to use this intervention, and it is feasible to implement, it was well tolerated, and it had no adverse effects. However, it was not effective in reducing indices of muscle damage, potentially highlighting the necessity of a loading phase with TCs, though the mechanisms are not fully elucidated as it was not in the scope of this study.

## Figures and Tables

**Table 1:** Participant characteristics (mean ± SD).

Group	Sex (*n*)	Age (Years)	Height (cm)	Mass (kg)
TC	Males (7)	22 ± 1	178 ± 5	87 ± 20
	Females (5)	23 ± 3	170 ± 5	68 ± 8
Placebo	Males (5)	23 ± 3	181 ± 6	68 ± 14
	Females (5)	26 ± 4	159 ± 7	60 ± 13

**Table 2:** Summary of other indirect markers of muscle damage to the elbow flexors.

		Pre	24	48	72
Pain Pressure Threshold (N) *	TC	35 ± 14	20 ± 8	19 ± 8	21 ± 7
	PLC	28 ± 10	20 ± 7	18 ± 6	19 ± 7
95% CI		28.93–44.24	27.12–51.13	19.59–49.59	−44.24–28.93
Limb Girth (mm) *	TC	322 ± 58	326 ± 58	325 ± 56	326 ± 56
	PLC	312 ± 44	316 ± 45	317 ± 44	316 ± 46
95% CI		−0.59–−0.17	−0.62–−0.10	−0.68–−0.01	0.17–0.59
Range of Motion (%) *	TC	100 ± 0	90 ± 4	88 ± 10	89 ± 11
	PLC	100 ± 0	93 ± 3	91 ± 6	89 ± 12
95% CI		7.37–11.88	7.71–16.06	6.75–17.25	−11.89–−7.37

* Indicates a significant time effect (*p* < 0.05). TC *n* = 12 and placebo *n* = 10.

## Data Availability

The data are kept on the university’s secure server in line with UK law relating to the General Data Protection Regulations and the university’s Research Data Management Policy. Requests for data should be sent to the corresponding author.
